# Comparison of next‐generation sequencing and cobas EGFR mutation test v2 in detecting *EGFR* mutations

**DOI:** 10.1111/1759-7714.14685

**Published:** 2022-10-06

**Authors:** Shuji Murakami, Tomoyuki Yokose, Kanako Shinada, Testuya Isaka, Kengo Katakura, Ryouta Ushio, Tetsuro Kondo, Terufumi Kato, Hiroyuki Ito, Haruhiro Saito

**Affiliations:** ^1^ Department of Thoracic Oncology Kanagawa Cancer Center Yokohama Japan; ^2^ Department of Pathology Kanagawa Cancer Center Yokohama Japan

**Keywords:** cobas EGFR mutation test v2, epidermal growth factor receptor gene (EGFR) mutation, next‐generation sequencing, non‐small cell lung cancer, oncomine dx target test

## Abstract

**Background:**

As the number of genetic mutations that must be tested increases, the Oncomine Dx Target test (ODxTT), which can simultaneously detect multiple cancer‐related genes is becoming the main test used in preference to single‐molecule testing. In this study, we evaluated the performance of ODxTT and cobas EGFR mutation test v2 (cobas EGFR), one of the single‐molecule tests, in detecting *EGFR* mutations.

**Methods:**

Samples from 211 patients diagnosed with NS‐NSCLC were tested simultaneously or sequentially with the cobas EGFR mutation test and ODxTT. We compared the success and detection rates of both tests and evaluated their equivalence by determining the concordance rate and k‐coefficient of both tests.

**Results:**

The success rate in detecting *EGFR* mutations was 95.7% for ODxTT and 100% for cobas EGFR. *EGFR* mutations were detected in 26.5% of samples with ODxTT and in 28.0% with cobas EGFR. For the 200 samples successfully analyzed with both tests, the concordance rate and k‐coefficient were 97.5% and 0.938, respectively. ODxTT failed to detect two exon 19 deletion mutations (p.E746_P753delinsVS and p.E746_P753delinsLS), and cobas EGFR failed to detect three instances of an exon 19 deletion (p.L747_P753delinsS), L861R, and an exon 20 insertion.

**Discussion:**

The success rate of ODxTT is slightly inferior to that of cobas EGFR. ODxTT shared a high concordance rate and k‐coefficient with cobas EGFR in detecting *EGFR* mutations, but discordant results between the two tests were observed in a few cases, mainly due to the difference of detectable EGFR variants.

## Graphical abstract

The success rate of oncomine Dx target test (ODxTT) is slightly inferior to that of cobas EGFR. The performance of ODxTT in detecting *EGFR* mutations is similar to that of cobas EGFR. Discordant results between the two tests were observed in a few cases, mainly because the two tests differ in their ability to detect EGFR variants. We need to understand the limitations of each companion diagnostic method, and even when a companion diagnostic test detects no actionable gene mutations, an NGS analysis, such as with a CGP method, should be considered, especially in patients who are likely to have some genetic mutations, such as young people and nonsmokers.

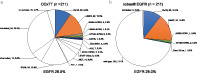

## INTRODUCTION

Non‐small cell lung cancer (NSCLC) is diagnosed at an advanced clinical stage in a large proportion of patients, entailing a poor clinical outcome. These patients are candidates for systemic chemotherapy, which may improve their survival and quality of life, but chemotherapy is still considered palliative. Since the introduction of targeted therapies for NSCLC, which were developed to block aberrant oncogenic signaling a decade ago, these therapies have opened a new era in the management of advanced NSCLC, improving survival outcomes.^1^ Targeting oncogenic driver mutations, such as sensitizing mutations in the epidermal growth factor receptor gene (EGFR)^2^, ^3^ and fusions of echinoderm microtubule‐associated protein‐like 4 (EML4) and anaplastic lymphoma kinase (ALK), rearrangements of the ROS proto‐oncogene 1 (ROS1), the BRAF V600E mutation, and the MET exon 14 skipping, has significantly improved the prognosis of advanced NSCLC, with higher response rates and longer progression‐free survival compared with those achieved with conventional cytotoxic chemotherapy. These therapies have been approved for clinical use for NSCLC.^4^, ^5^, ^6^, ^7^, ^8^, ^9^ Among the genetic mutations with prognostic value, EGFR mutations are most frequently detected, occurring in 45% of patients with advanced non‐squamous NSCLC (NS‐NSCLC) in Japan.^10^ Several clinical trials evaluating EGFR‐tyrosine kinase inhibitors (TKIs) have been conducted, and the median overall survival (OS) of patients with EGFR‐activating mutations treated with EGFR‐TKI has reached 4–5 years in recent clinical trials.^11^, ^12^, ^13^

Conventionally, platinum‐based chemotherapy has been the basic treatment for NSCLC patients without driver mutations. However, its efficacy had reached a plateau, with a median OS of 1 year.^14^, ^15^, ^16^, ^17^ Recently, the development of immune checkpoint inhibitors (ICIs) has dramatically changed the treatment of advanced NSCLC. ICIs provide long‐term survival in some patients with advanced NSCLC, and ICI monotherapy or the combination of ICIs with cytotoxic chemotherapy has become the standard treatment for advanced NSCLC without driver mutations.^18^, ^19^ The response to ICI monotherapy is about 20% in unselected NS‐NSCLC patients,^20^ but the effect is limited in patients with some driver mutations,^20^, ^21^, ^22^ such as those in EGFR.

Therefore, molecular biomarker testing has become crucial when decisions on treatment are made, especially in patients with advanced NS‐NSCLC, because the efficacy of targeted therapies can be predicted. Conventionally, the real‐time reverse transcription–polymerase chain reaction (RT–qPCR)‐based cobas EGFR mutation test v2 (cobas EGFR: Roche Molecular Systems), which can identify 42 different *EGFR* mutations in exons 18, 19, 20, and 21, has been widely used as a single companion diagnostic test for therapies with EGFR‐TKIs. However, as the number of genetic mutations that must be tested increases, the ability of a companion molecular test to screen each genetic mutation simultaneously or sequentially is approaching its limit.

Next‐generation sequencing (NGS) is a recently developed, massive, parallel, large‐scale sequencing technology that is becoming a key technique for the simultaneous screening of multiple cancer‐related genes.^23^ Oncomine Dx target test (ODxTT; Thermo Fisher Scientific), which simultaneously evaluates 46 cancer‐related genes, was the first NGS panel for NSCLC testing approved by the US Food and Drug Administration, in June 2017.^24^ In daily clinical practice in Japan, ODxTT is often performed if a sufficient amount of tumor tissue can be collected. NGS is a promising technology for the simultaneous detection of multiple genes, but it has several limitations. It requires specimens containing nucleic acids of sufficient quality and quantity. Poor quality or too little tumor sample can cause the failure of an NGS analysis. For ODxTT, the estimated tumor content of the biopsy sample is recommended to be >30% of the total cells, which is higher than the tumor content of 5% recommended for cobas EGFR. We also previously reported that a sufficient amount of tissue is required to successfully exploit the NGS technology with ODxTT.^25^, ^26^ ODxTT is based on amplicon sequencing, and it is a highly targeted approach to the analysis of genetic variations using PCR, with sets of primers for exons or hotspots in selected genes. Therefore, ODxTT cannot detect untargeted gene mutations, but is designed to detect various EGFR mutations in exons 3, 7, 12, 15,18, 19, 20, and 21 (Table S1).

In this study, we evaluated the consistency of cobas EGFR and ODxTT in detecting *EGFR* mutations and the effect of the *EGFR* mutation frequencies on the number of variants detected with the two methods.

## METHODS

### Patients

The study participants included 211 patients who were pathologically diagnosed with NS‐NSCLC, and were subjected for ODxTT testing between September 2019 and August 2021 at the Kanagawa Cancer Center Hospital, Japan. The tissue sampling procedures included transbronchial biopsy (TBB) with endobronchial ultrasonography with a guide sheath (EBUS‐GS), endobronchial biopsy (EBB) with direct‐vision forceps, endobronchial ultrasound‐guided transbronchial needle aspiration (EBUS‐TBNA), computed tomography (CT)‐guided biopsy, and surgical resection from the lung or other sites. When the tumor samples were judged by the pathologist to contain sufficient cancer cells, a biomarker analysis was performed with ODxTT, regardless of the patient characteristics or clinical background. Samples in which only ODxTT was performed were retrospectively tested with cobas EGFR, using the residual specimens originally used for ODxTT. After April 2021, ODxTT and cobas EGFR were performed simultaneously on the same specimens. Cobas EGFR was performed on all but two samples for which there was no residual specimen. We retrospectively reviewed the medical records of patients and evaluated the patient characteristics, sampling methods, staging, and the results of genetic tests. When *EGFR* mutations detected with ODxTT and cobas EGFR were discordant, we reviewed the clinical course of treatment and the treatment outcome, and the results of a genetic analysis with oncomine comprehensive assay v3 (OCA v3; Thermo Fisher Scientific) using fresh‐frozen tissues, which was performed in a lung cancer genomic screening project for individualized medicine in Japan (LC‐SCRUM) (UMIN ID: UMIN000010234). We obtained ethical approval for the study from the Kanagawa Cancer Center Hospital Japan (2019EKI‐48), and patient confidentiality was maintained. Written informed consent was obtained from all subjects when undergoing ODxTT.

### Tumor specimens and genetic analysis of *EGFR* mutations

The tumor samples were fixed in 10% neutral‐buffered formalin solution for 6–24 h, embedded in paraffin wax, and processed for histopathological examination with routine histology techniques. The histological diagnosis was made by pathologists according to the World Health Organization (WHO) classification of lung tumors.^27^

After the pathological diagnosis of NSCLC, we routinely screened for therapy‐predictive biomarkers. We assessed three pathological factors that potentially influence the success rate of NGS analysis in clinical practice: the tissue surface area, the tumor cell count, and the tumor content ratio. In general, the estimated tumor content in a biopsy sample is recommended to be >30% of the total cells for ODxTT. Since January 2020, we added to our local submission criteria a tissue surface area of ≥1 mm^2^. When there was sufficient tissue, 10–30 glass slides adhered with 5 μm thickened tissue pieces were prepared for ODxTT from each tumor sample, depending on the tumor cell count and tissue area per slide. More five sections of 10 μm thickness were simultaneously or sequentially cut from each specimen for cobas EGFR. These samples were submitted to SRL Laboratories, a Japanese commercial laboratory, at which ODxTT and cobas EGFR testing was performed.

In this study, we define analytical “success” as samples successfully reported as positive or negative for *EGFR* mutations and BRAF V600E with DNA sequencing and for ALK and ROS1 with RNA sequencing using ODxTT, and analysis “failure” as samples reported as “no call” or “invalid” for these four driver mutations.

### Statistical analysis

The patient background, analytical success rate, and detection rate for *EGFR* mutations with each test were analyzed for all enrolled patients. The analytical success and detection rates for *EGFR* mutations achieved with ODxTT and cobas EGFR were compared with Pearson's χ^2^ test. Statistical significance was defined as a *p*‐value of <0.05. The equivalence between ODxTT and cobas EGFR in detecting *EGFR* mutations was evaluated with concordance rates and the κ‐coefficient. The concordance rates and κ‐coefficients were determined for samples successfully analyzed with both ODxTT and cobas EGFR. A κ‐coefficient of >0.80 was considered to indicate excellent agreement between the methods.

## RESULTS

### Patient characteristics

A total of 211 patients who were pathologically diagnosed with NS‐NSCLC and analyzed with ODxTT were reviewed in this study. In addition, there were 177 patients for whom only a single test was performed over the same period. The patient characteristics are summarized in Table 1. The mean age of patients was 71 years. Of the 211 patients, 140 (66.4%) were men, and 150 (71.1%) were smokers. The distribution by stage was as follows: stage I, 23 patients; stage II, 27 patients; stage III, 42 patients; stage IV, 111 patients; and postoperative recurrence, eight patients. Tumor tissues were sampled with large EBUS‐GS (FB‐231D; Olympus Medical Systems; *n* = 89), small EBUS‐GS (FB‐233D; Olympus Medical Systems; *n* = 12), EBB (n = 7), EBUS‐TBNA (*n* = 25), CT‐guided biopsy (*n* = 10), or lung surgical resection (*n* = 47). Most patients were diagnosed with adenocarcinoma (83.4%), followed by “not otherwise specified” (NOS; 8.5%) and NSCLC (5.3%). The success rate of DNA sequencing for ODxTT was 95.7% (202/211), RNA sequencing was 97.6% (206/211), and both types of sequencing was 94.7% (200/211) across all patients.

**TABLE 1:** Patient characteristics

		ODxTT	
		Success	Failure
Characteristic	(*n* = 211)	(*n* = 200)	(*n* = 11)
Median age (range)	71 (38–90)	71 (38–90)	69 (49–80)
Sex (male/female)	140/71	135/67	6/5
Stage (I/II/III/IV/r)	23/27/42/111/8	22/27/40/104/7	1/0/2/7/1
Smoker/nonsmoker	150/61	144/57	7/4
Smoking index	560 (0–2940)	570 (0–2940)	136 (0–2000)
Tissue sample			
Large EBUS‐GS TBLB	89	86	3
Small EBUS‐GS TBLB	12	6	6
EBB	7	6	1
EBUS‐TBNA	25	25	0
CT‐guided biopsy	10	10	0
Lung surgery	47	47	0
Other	15	14	1
Histology			
Adenocarcinoma	176	166	10
NOS	18	18	0
NSCLC	11	11	0
Pleomorphic	4	4	0
Adenosquamous	2	1	1

Abbreviations: EBB, endobronchial biopsy; EBUS‐GS, endobronchial ultrasonography with a guide sheath; EBUS‐TBNA, endobronchial ultrasound‐guided transbronchial needle aspiration; NOS, not otherwise specified; ODxTT, oncomine Dx target test; r, recurrence; TBLB, transbronchial lung biopsy.

### Test success rate and frequency of driver mutations from each genetic analysis

The success rate of *EGFR* mutation detection for ODxTT was 95.7% (202/211); two samples were diagnosed as “insufficient quantity” and seven were diagnosed as “no‐call”. There was insufficient sample left to perform cobas EGFR testing in two patients, but cobas EGFR was performed in the remaining 209 patients. The cobas EGFR test was successfully analyzed in all patients, with no failure (*p* = 0.0025).

**FIGURE 1:**
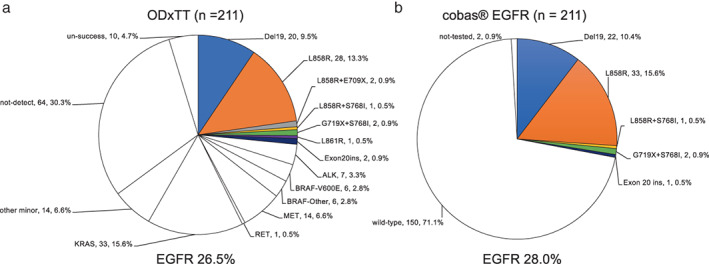
Frequency of driver mutations detected with ODxTT and cobas EGFR (a) detection rate of driver mutations with ODxTT. (b) Detection rate of *EGFR* mutations with cobas EGFR

The frequency of *EGFR* mutations detected with each test was 26.5% for ODxTT and 28.0% for cobas EGFR, across all patients (*p* = 0.7429) (Figure 1a,b). *EGFR* mutations were only detected in adenocarcinomas. When limited to adenocarcinomas, the frequency of *EGFR* mutations detected was 31.8% (56/176) with ODxTT and 33.5% (59/176) with cobas EGFR (*p* = 0.7332). Of the 59 samples in which *EGFR* mutations were detected with cobas EGFR, none were detected in four samples with ODxTT due to “no‐call”. Compound mutations, defined as double mutations in the *EGFR* kinase domain, were detected in 8.9% (5/56) of samples with ODxTT and in 5.1% (3/59) of samples with cobas EGFR (*p* = 0.418).

### Agreement of cobas EGFR and ODxTT in the detection of *EGFR* mutations

Among the 200 samples analyzed with both tests, discordant results, in which an *EGFR* mutation was only detected with only one assay, were obtained for five samples. The concordance rate and κ‐coefficient were 97.5% (95% confidence interval [CI]: 94.3%–99.2%) and 0.938 (95% CI: 0.859–0.971), respectively (Table 2). ODxTT failed to detect two exon 19 deletions (p.E746_P753delinsVS and p.E746_P753delinsLS), which were confirmed with OCA (Table 3). In contrast, cobas EGFR failed to detect three instances of an exon 19 deletion (p.L747_P753delinsS), L861R, and an exon 20 insertion (p.P772_H733insHV). Two compound *EGFR* mutations were detected only with ODxTT, and both involved mutations L858R and E709X.

**TABLE 2:** Analysis of agreement between ODxTT and cobas EGFR in detecting *EGFR* mutations

		Cobas EGFR			
		Present	Negative	No sample	Total
ODxTT	Present	53	3	0	56
	Negative	2	142	2	146
	Not evaluable	4	5	0	9
	Total	59	150	2	211

Abbreviations: cobas EGFR, cobas EGFR mutation test v2; ODxTT, Oncomine Dx target test.

**TABLE 3:** Patient characteristics and outcomes of patients with discrepant results

ODxTT	Allele frequency	Cobas EGFR	OCA	Age	Sex	Stage	EGFR‐TKI	Treatment	Response	TTF
Not detected	‐	Del 19	Del 19: pE746‐P753 > VS	82	F	IVA	Osimertinib	Second‐line	PR	17 m+
Not detected	‐	Del 19	Del 19: pE746‐P753 > LS	71	M	IVB	Osimertinib	First‐line	PR	12 m+
Del 19: p.L747‐P753 > S	0.075	not detected	Del 19: p.L747‐P753 > S	82	F	r	Osimertinib	First‐line	PR	8 m+
L861R	0.23	not detected	‐	74	M	IIIA	‐	‐	‐	‐
Exon 20ins: p.P772‐H773 insHV	0.285	not detected	‐	69	M	r	‐	‐	‐	‐
L858R, E709G	0.173, 0.174	L858R	‐	71	F	IVB	‐	‐	‐	‐
L858R, E709K	0.37, 0.349	L858R	‐	85	F	IVA	Osimertinib	First‐line	PR	5 m+

Abbreviations: cobas EGFR, cobas EGFR mutation test v2; Del 19, exon 19 deletion; F, female; M, male; m, months, OCA, oncomine comprehensive assay v3; ODxTT, oncomine Dx target test; PR, partial response; r, recurrence; TTF, time to treatment failure.

Of the three patients in whom discordant results involved an exon 19 deletion, an EGFR‐TKI (osimertinib) was administered to two patients as first‐line treatment and was administered to the other patient as a second‐line therapy, and these three patients showed tumor shrinkage. One patient with an EGFR exon 20 insertion was treated with cytotoxic chemotherapy with an ICI. One patient with the L858R and E709G mutations was transferred to another hospital. The other patient with the L858R and E709K mutations was treated with osimertinib and displayed tumor shrinkage.

## DISCUSSION

In this study, we evaluated the performance of ODxTT and cobas EGFR in detecting *EGFR* mutations, which is essential for the assessment of advanced NS‐NSCLC patients, because the frequency of EGFR mutations associated with this disease is high. Cobas EGFR is a highly sensitive and reliable test that is commonly used as a single test for detecting *EGFR* mutations. Therefore, it is important to know whether ODxTT, the first approved NGS panel testing in Japan, is inferior to cobas EGFR. In this comparison, we detected some differences in the success rates of ODxTT and cobas EGFR, but the two tests showed high concordance on the samples successfully analyzed with both tests. However, discordant results were observed in several cases, although EGFR‐TKI caused tumor shrinkage, even in some discordant cases.

Genotyping tumors at the time of diagnosis is essential for determining the optimal first‐line treatment for advanced NSCLC. Among the oncogenic driver mutations of this cancer, those in EGFR are among the most important genomic mutations and their detection is essential, especially in female and nonsmoker patients, who frequently carry them.^28^, ^29^ In the BRAVE study, a multicenter retrospective observational study of single biomarker testing in advanced NSCLC patients in Japan, the proportions of patients who underwent individual biomarker testing for EGFR, ALK, and ROS1 were 97.5, 88.1, and 67.3%, respectively.^30^ A previous study showed that as the number of single biomarker tests performed for each driver mutation increased, the number of single tests that could be ordered decreased accordingly.^31^ To solve these clinical problems, NGS testing, which can analyze multiple genes simultaneously, must be widely accessible.

Both ODxTT and cobas EGFR are widely used in clinical practice for the detection of EGFR mutations in NSCLC tumor samples, but using these two tests simultaneously is not permitted by the Japanese national healthcare policy. Previously, the cobas EGFR test was mainly used, but it has been replaced by the NGS test, which can simultaneously detect *EGFR* mutations and other driver mutations. In general, compared with NGS, PCR‐based tests have few failures, the results are obtained rapidly, and even small samples can be tested. Therefore, depending on the patient's background, cobas EGFR may be prioritized in daily clinical practice. The success rate when screening for genomic changes with cobas EGFR was 100%, which was higher than the success rate with ODxTT (95.7%) in present study. An NGS analysis, which can detect a large number of genes, requires a large amount of tumor sample, and the success rates of NGS analyses based on small samples can be low.^32^ In our previous study, we suggested that taking larger samples increases the success rate of mutation detection with ODxTT and that the success rate varies according to the biopsy device used.^25^, ^26^ In the present study, 10 of the 11 patients who were unsuccessfully tested with ODxTT were sampled with bronchoscopy, and six of these were sampled using small‐forceps with a small EBUS‐GS. These findings indicate that clinicians must strive to ensure that a sufficient amount of tumor cells is retrieved for successful analysis with ODxTT.

In this study, the detection rate for *EGFR* mutations was 26.5% with ODxTT and 28.0% with cobas EGFR. These frequencies are rather low compared with the frequency of ~45% previously reported in Japanese NSCLC patients.^10^ In a retrospective study of 390 patients with adenocarcinoma whose genetic changes were analyzed with ODxTT (West Japan Oncology Group [WJOG] 1309 L),^33^ the frequency of *EGFR* mutations was 29.5%, similar to our results. This trend is attributable to the large number of males (66.4%) and smokers (71.1%) enrolled in our study. Moreover, ODxTT was only performed for patients with sufficient tumor tissue to meet the submission criteria, which may have affected the selection bias. High concordance was observed between ODxTT and cobas EGFR in detecting *EGFR* mutations among the patients successfully analyzed with both tests, but the results for five samples were discordant. Cobas EGFR missed one mutation, a deletion in exon 19, probably because the percentage of mutated DNA in this sample was low (7.5%), and the cobas EGFR assay is only reliable when the sample contains >5% mutant DNA. In the remaining four cases, ODxTT failed to detect two mutations, both deletions in exon 19 (p.E746_P753delinsVS and p.E746_p753delinsLS), which is not detectable variant type by ODxTT. Conversely, cobas EGFR failed to detect mutation L861R in two samples and an insertion in exon 20 (p.P772_H733insV), which are not detectable variant by cobas EGFR. Cobas EGFR was unable to detect E709X, which was detected with ODxTT in a compound mutation with L858R in two samples in the present study. Importantly, the third‐generation EGFR‐TKI osimertinib was used in three patients with discordant results for exon 19 deletions, all of whom showed a partial clinical response.

Currently, two gene‐panel tests, FoundationOne CDx (F1CDx) and the NCC OncoPanel, in addition to ODxTT, have been approved in Japan.^34^ ODxTT is categorized as a hot‐spot panel test based on amplicon sequences, and amplifies each targeted site with PCR and primers spanning part of the coding region. Therefore, ODxTT can be used with a small amount of DNA or RNA but can only detect mutations at the targeted mutational hotspots. F1CDx and the NCC OncoPanel have been approved as comprehensive genome profile tests, which use the hybrid capture method, and can detect mutations, amplifications, and homozygous deletions in the entire coding regions of the targeted genes, together with rearrangements of the targeted oncogenes included in each panel. Therefore, using the hybrid capture method, F1CDx and the NCC OncoPanel can detect rare variants that cannot be detected with hot‐spot panel tests, including ODxTT and cobas EGFR. In fact, *EGFR* mutations were the most frequently identified actionable genetic aberrations using the NCC OncoPanel, even in patients with NSCLC, in whom no *EGFR* mutations or ALK fusions were detected using single companion diagnostics.^35^ It is noteworthy that these patients in whom *EGFR* mutations were detected with the NCC OncoPanel were treated with EGFR‐TKIs, with demonstrable therapeutic effects.^35^, ^36^ Thus, F1CDx and the NCC OncoPanel were able to detect EGFR variants that cannot be detected by ODxTT and cobas EGFR, leading to a reduction in the number of *EGFR* mutation‐positive patients left undetected, giving patients the chance to receive EGFR‐TKIs. Based on these facts, even when a companion diagnostic test detects no actionable gene mutations, an NGS analysis, such as with a CGP method, highly recommended, especially in patients who are likely to have some genetic mutations, such as young people and nonsmokers.

The present study had several limitations. First, there was potential for selection bias because the study was conducted at a single institution and only patients for whom ODxTT was performed were included. ODxTT was mainly performed on tumor samples that met the submission criteria of our institution. Patients who were diagnosed with cytology alone or for whom there was insufficient tumor sample for ODxTT were not included in the study. Therefore, the frequency of *EGFR* mutations in this study referred to a selected cohort of patients, rather than all NS‐NSCLC patients. Secondly, a relatively small sample of patients with *EGFR* mutations was used to evaluate the rate of discrepancies between the two tests in detecting *EGFR* mutations, so the false‐negative rate was not accurate. Furthermore, neither test detected all of the variants of EGFR, so both tests produced false‐negative results for some rare EGFR variants. Finally, because we evaluated only *EGFR* mutations, the consistency between ODxTT and a single test for other mutations has still to be examined.

In conclusion, the success rate of ODxTT is acceptable in clinical practice, but slightly inferior to that of cobas EGFR. However, the performance of ODxTT in detecting *EGFR* mutations is similar to that of cobas EGFR. Nonetheless, discordant results between the two tests were observed in a few cases, mainly because the two tests differ in their ability to detect EGFR variants. Therefore, the advantages and limitations of each test must be clarified to ensure that genomic testing methods are used properly.

## CONFLICT OF INTEREST

Shuji Murakami reports personal fees from AstraZeneca, Chugai Pharmaceutical, Boehringer Ingelheim, Taiho Pharmaceutical, and Ono Pharmaceutical.

Terufumi Kato reports grants and personal fees from MSD, Novartis, Ono Pharmaceutical, Pfizer, and Taiho Pharmaceutical, personal fees from Daiichi Sankyo, F. Hoffmann‐La Roche, Nippon Kayaku, Nitto Denko, Shionogi Pharmaceutical, Sumitomo Dainippon, and Takeda, and grants from Astellas, Kyorin, Kyowa Kirin, and Regeneron.

Haruhiro Saito reports grants from Chugai Pharmaceutical and AstraZeneca, and personal fees from Ono Pharmaceutical, Nippon Boehringer Ingelheim, MSD, and Novartis Pharma.

The other authors report no conflicts of interest.

## Supporting information


**Table S1.** Detectable EGFR mutations from Cobas EGFR ver2.0 and Oncomine DxTT.
