# Supplementary material for: Comparison of next‐generation sequencing and cobas EGFR mutation test v2 in detecting EGFR mutations
Source: Thorac Cancer. 2022 Oct 6;13(22):3217–24. doi: 10.1111/1759-7714.14685 (PMC9663664; doi:10.1111/1759-7714.14685)
Supplement: Supplementary file 1 — Table S1. Detectable EGFR mutations from Cobas EGFR ver2.0 and Oncomine DxTT. [file TCA-13-3217-s001.docx]

Supplemental Table1 : detectable EGFR mutations from Cobas EGFR ver2.0 and Oncomine DxTT

| Exon region | COSMIC ID | amino acid substitution | Cobas EGFR ver2.0 | Oncomine DxTT |
| --- | --- | --- | --- | --- |
| Exon3 | COSM6939064 | p.R108G |  | 〇 |
|  | COSM5576118 | p.R108K |  | 〇 |
| Exon7 | COSM6356510 | p.A289T |  | 〇 |
|  | COSM21685 | p.A289D |  | 〇 |
|  | COSM21687 | p.A289V |  | 〇 |
| Exon12 | COSM236671 | p.S492R |  | 〇 |
|  | COSM236670 | p.S492R |  | 〇 |
| Exon15 | COSM3412196 | p.G598A |  | 〇 |
|  | COSM21690 | p.G598V |  | 〇 |
| Exon18 | COSM12988 | p.E709K |  | 〇 |
|  | COSM13427 | p.E709A |  | 〇 |
|  | COSM13009 | p.E709G |  | 〇 |
|  | COSM12371 | p.E709V |  | 〇 |
|  | COSM1169617 | p.L718V |  | 〇 |
|  | COSM6503269 | p.L718Q |  | 〇 |
|  | COSM6252 | p.G719S | 〇 | 〇 |
|  | COSM6253 | p.G719C | 〇 | 〇 |
|  | COSM18425 | p.G719D |  | 〇 |
|  | COSM6239 | p.G719A | 〇 | 〇 |
|  | COSM13979 | p.G724S |  | 〇 |
| Exon19 | COSM87245 | p.I744T |  | 〇 |
|  | COSM26038 | p.K745_E749del | 〇 | 〇 |
|  | COSM1190791 | p.K745_A750delinsT |  | 〇 |
|  | COSM28517 | p.E746_E749del |  | 〇 |
|  | COSM13550 | p.E746_A750delinsIP | 〇 |  |
|  | COSM6223 | p.E746_A750del | 〇 | 〇 |
|  | COSM13552 | p.E746_T751delinsIP | 〇 |  |
|  | COSM13551 | p.E746_T751delinsI | 〇 | 〇 |
|  | COSM12385 | p.E746_S752delinsI | 〇 |  |
|  | COSM6225 | p.E746_A750del | 〇 | 〇 |
|  | COSM12728 | p.E746_T751del | 〇 | 〇 |
|  | COSM12678 | p.E746_T751delinsA | 〇 | 〇 |
|  | COSM12386 | p.E746_T751delinsV | 〇 |  |
|  | COSM12416 | p.E746_T751delinsVA | 〇 | 〇 |
|  | COSM12367 | p.E746_S752delinsA | 〇 |  |
|  | COSM12384 | p.E746_S752delinsV | 〇 | 〇 |
|  | COSM18427 | p.E746_P753delinsVS | 〇 |  |
|  | COSM12422 | p.L747_A750delinsP | 〇 | 〇 |
|  | COSM12419 | p.L747_T751delinsQ | 〇 | 〇 |
|  | COSM6220 | p.E746_S752delinsD | 〇 | 〇 |
|  | COSM6218 | p.L747_E749del | 〇 | 〇 |
|  | COSM12382 | p.L747_A750delinsP | 〇 | 〇 |
|  | COSM12383 | p.L747_T751delinsP | 〇 | 〇 |
|  | COSM6255 | p.L747_S752del | 〇 | 〇 |
|  | COSM12403 | p.L747_S752delinsQ | 〇 |  |
|  | COSM12387 | p.L747_P753delinsQ | 〇 | 〇 |
|  | COSM6210 | p.L747_T751delinsS | 〇 | 〇 |
|  | COSM12369 | p.L747_T751del | 〇 | 〇 |
|  | COSM12370 | p.L747_P753delinsS | 〇 | 〇 |
|  | COSM13556 | p.S752_I759del | 〇 |  |
| Exon20 | COSM26720 | p.A763_Y764insFQEA |  | 〇 |
|  | COSM12376 | p.A767_V769dup | 〇 | 〇 |
|  | COSM1651740 | p.A767_S768insYVM |  | 〇 |
|  | COSM12425 | p.A767_S768insTLA |  | 〇 |
|  | COSM13559 | p.A767_S768insIA |  | 〇 |
|  | COSM1651741 | p.V769_D770insANV |  | 〇 |
|  | COSM20884 | p.M766_A767insASV |  | 〇 |
|  | COSM6984779 | p.S768_V769delinsIleL |  | 〇 |
|  | COSM85750 | p.S768_V769delinsIleL |  | 〇 |
|  | COSM13428 | p.S768_D770dup | 〇 | 〇 |
|  | COSM6241 | p.S768I | 〇 | 〇 |
|  | COSM20885 | p.V769_N771dup |  | 〇 |
|  | COSM6506514 | p.V769dup |  | 〇 |
|  | COSM28638 | p.V769_D770insMASVD |  | 〇 |
|  | COSM12379 | p.V769_D770insCV |  | 〇 |
|  | COSM6983510 | p.D770delinsGTH |  | 〇 |
|  | COSM18429 | p.V769_D770insGSV |  | 〇 |
|  | COSM1235344 | p.V769_D770insGSV |  | 〇 |
|  | COSM18430 | p.V769_D770insGVV |  | 〇 |
|  | COSM12427 | p.D770delinsGY |  | 〇 |
|  | COSM1651745 | p.D770_P772dup |  | 〇 |
|  | COSM13558 | p.A767_V769dup | 〇 | 〇 |
|  | COSM1651742 | p.V769_D770insERG |  | 〇 |
|  | COSM12737 | p.D770_N771delinsAGG |  | 〇 |
|  | COSM4970107 | p.D770_N771insQRG |  | 〇 |
|  | COSM20886 | p.D770_N771insAPW |  | 〇 |
|  | COSM13004 | p.D770_N771insG |  | 〇 |
|  | COSM1238029 | p.D770_N771insGT |  | 〇 |
|  | COSM22955 | p.D770_N771insGD |  | 〇 |
|  | COSM85795 | p.D770_N771insGD |  | 〇 |
|  | COSM48921 | p.D770_N771insGL |  | 〇 |
|  | COSM655155 | p.D770_N771insGF |  | 〇 |
|  | COSM12378 | p.D770_N771insG | 〇 | 〇 |
|  | COSM1238030 | p.D770_N771insY |  | 〇 |
|  | COSM6962256 | p.A763_Y764insYVMASVD |  | 〇 |
|  | COSM53189 | p.N771delinsGY |  | 〇 |
|  | COSM18431 | p.N771delinsGF |  | 〇 |
|  | COSM22946 | p.N771delinsTH |  | 〇 |
|  | COSM5023008 | p.D770_N771insT |  | 〇 |
|  | COSM6920147 | p.N771delinsSTH |  | 〇 |
|  | COSM1651743 | p.D770_N771insSVE |  | 〇 |
|  | COSM24434 | p.N771delinsSH |  | 〇 |
|  | COSM1651744 | p.N771delinsSGH |  | 〇 |
|  | COSM26719 | p.D770_N771insMATP |  | 〇 |
|  | COSM13003 | p.N771dup |  | 〇 |
|  | COSM12381 | p.N771_H773dup |  | 〇 |
|  | COSM5023007 | p.N771delinsVH |  | 〇 |
|  | COSM6438147 | p.N771delinsKL |  | 〇 |
|  | ー | p.V769_D770insDK |  | 〇 |
|  | COSM1238031 | p.N771_P772insH |  | 〇 |
|  | ー | p.N771delinsKG |  | 〇 |
|  | ー | p.N771_P772insT |  | 〇 |
|  | COSM6922328 | p.N771_P772insV |  | 〇 |
|  | ー | p.N771_P772insL |  | 〇 |
|  | COSM6931207 | p.N771_P772insHH |  | 〇 |
|  | COSM166390 | p.N771_P772insRH |  | 〇 |
|  | ー | p.N771_P772insL |  | 〇 |
|  | COSM12380 | p.P772_H773dup |  | 〇 |
|  | COSM48923 | p.P772_H773insTHP |  | 〇 |
|  | COSM6845099 | p.V769_D770insDNP |  | 〇 |
|  | COSM1238028 | p.H773_V774insAH |  | 〇 |
|  | ー | p.P772_H773insHPHP |  | 〇 |
|  | ー | p.D770_N771insNP |  | 〇 |
|  | COSM6977296 | p.D770_N771insNPTP |  | 〇 |
|  | ー | p.D770_N771insNPG |  | 〇 |
|  | ー | p.D770_N771insNPG |  | 〇 |
|  | ー | p.S768_V769insVDNP |  | 〇 |
|  | COSM255205 | p.P772_H773insV |  | 〇 |
|  | COSM18432 | p.H773_V774dup |  | 〇 |
|  | COSM12388 | p.P772_H773insTP |  | 〇 |
|  | ー | p.H773delinsNPY |  | 〇 |
|  | ー | p.D770_N771insNPP |  | 〇 |
|  | COSM1735761 | p.H773delinsPNPY |  | 〇 |
|  | COSM12377 | p.H773dup | 〇 | 〇 |
|  | COSM22948 | p.H773_V774dup |  | 〇 |
|  | COSM5023006 | p.H773_V774insNH |  | 〇 |
|  | COSM3727813 | p.H773_V774insTQPP |  | 〇 |
|  | COSM131552 | p.H773_V774insQ |  | 〇 |
|  | COSM51544 | p.H773_V774insGNPH |  | 〇 |
|  | COSM4170223 | p.V774_C775insPR |  | 〇 |
|  | COSM6845098 | p.N771_P772insPHV |  | 〇 |
|  | COSM22954 | p.C775Y |  | 〇 |
|  | COSM6240 | p.T790M | 〇 | 〇 |
|  | ー | p.L792V |  | 〇 |
|  | COSM6493934 | p.L792H |  | 〇 |
|  | COSM20891 | p.G796S |  | 〇 |
|  | COSM6493935 | p.G796R |  | 〇 |
|  | ー | p.G796C |  | 〇 |
|  | COSM6493937 | p.C797S |  | 〇 |
|  | COSM5945664 | p.C797S |  | 〇 |
| Exon21 | COSM87246 | p.T854I |  | 〇 |
|  | COSM12366 | p.L858M |  | 〇 |
|  | COSM12429 | p.L858R | 〇 |  |
|  | COSM6224 | p.L858R | 〇 | 〇 |
|  | COSM6213 | p.L861Q | 〇 | 〇 |
|  | COSM12374 | p.L861R |  | 〇 |
